# Mammalian enamel maturation: Crystallographic changes prior to tooth eruption

**DOI:** 10.1371/journal.pone.0171424

**Published:** 2017-02-14

**Authors:** Anna Kallistová, Ivan Horáček, Miroslav Šlouf, Roman Skála, Michaela Fridrichová

**Affiliations:** 1 Institute of Geochemistry, Mineralogy and Mineral Resources, Faculty of Science, Charles University in Prague, Albertov 6, Czech Republic; 2 Institute of Geology of the CAS, v.v.i., Rozvojová 269, Prague 6, Czech Republic; 3 Department of Zoology, Faculty of Science, Charles University in Prague, Viničná 7, Czech Republic; 4 Institute of Macromolecular Chemistry of CAS v.v.i., Heyrovského náměstí 2, Prague 6, Czech Republic; Universiteit Gent, BELGIUM

## Abstract

Using the distal molar of a minipig as a model, we studied changes in the microstructural characteristics of apatite crystallites during enamel maturation (16-23 months of postnatal age), and their effects upon the mechanical properties of the enamel coat. The slow rate of tooth development in a pig model enabled us to reveal essential heterochronies in particular components of the maturation process. The maturation changes began along the enamel-dentine junction (EDJ) of the trigonid, spreading subsequently to the outer layers of the enamel coat to appear at the surface zone with a 2-month delay. Correspondingly, at the distal part of the tooth the timing of maturation processes is delayed by 3-5 month compared to the mesial part of the tooth. The early stage of enamel maturation (16-20 months), when the enamel coat is composed almost exclusively of radial prismatic enamel, is characterized by a gradual increase in crystallite thickness (by a mean monthly increment of 3.8 nm); and an increase in the prism width and thickness of crystals composed of elementary crystallites. The late stage of maturation (the last two months prior to tooth eruption), marked with the rapid appearance of the interprismatic matrix (IPM) during which the crystals densely infill spaces between prisms, is characterized by an abrupt decrease in microstrain and abrupt changes in the micromechanical properties of the enamel: a rapid increase in its ability to resist long-term load and its considerable hardening. The results suggest that in terms of crystallization dynamics the processes characterizing the early and late stage of mammalian enamel maturation represent distinct entities. In regards to common features with enamel formation in the tribosphenic molar we argue that the separation of these processes could be a common apomorphy of mammalian amelogenetic dynamics in general.

## Introduction

Tooth development is a highly organized and complex process of interactions between neural crest-derived ectomesenchyme and oral epithelium [1] proceeding under the control of specific signalling cascades [2–4] in recurrent steps common to all gnathostomes [5]. The development of a tooth terminates with the attaining of its final shape and the onset of the mineralization process at the late cap stage of tooth development [6]. There is just one mineral compound which forms all mineralized tissues of vertebrate bodies: the carbonated hydroxyapatite (CaP) [7, 8]. However, the two mineralized tissues composing adult teeth, dentine and enamel, differ essentially in amount of mineral compound, function and mechanisms of mineralization. Dentine produced by odontoblasts of mesenchymal papilla contains >20% matrix proteins, and its mineralization is organized by an active collagen scaffold formed by odontoblast processes and a large set of specific matrix proteins, distinct from those involved in enamel mineralization [6]. In contrast, mature enamel, the hardest tissue of the vertebrate body, is constituted by traces of organic material (roughly 4%) [6] such as proteins, amino acids and peptides [9–11] and its mineralization is organized by the molecular interaction of amorphous calcium matter and a limited set of specific proteins, both produced by a single layer of epithelial ameloblasts, i.e. with no further organic intervention [12]. The internal architecture of tooth enamel can be very complicated particularly in mammals, the group bearing extremely diversified diphyodont dentition with monophyodont multicuspidate molars and prismatic enamel. Enamel prisms, prolonged linear aggregates of densely packed parallel CaP crystallites, compose the driving factor uderlying the structural complexity of mammalian enamel. Their form and spatial organization are among the key dental characteristics of particular clades, a topic addressed by an enormous large number of comparative studies which have revealed quite detailed information on the functional relations, origin and phylogeny of mammalian prismatic patterns [13–16]. This holds true for enamel mineralization proper, the processes producing the final mechanical qualities of the adult tooth crown.

Amelogenesis, or enamel formation, is often reported to proceed in two distinct steps, the secretory and maturation stages, differing in the types of enamel-matrix proteins involved and the overall density of the mineral composition [17–21]. The secretory stage initiating histogenetic changes producing mature ameloblasts and the disappearance of the basal lamina of inner enamel epithelia is characterized by the secretion of enamel matrix proteins (EMP) into the extracellular space, where enamel mineral deposition takes place concurrently [22]. The essential components of EMP (amelogenin, ameloblastin, enamelin) are characterized by a conspicuously high rate of intrinsic disorder and a pronounced capacity to self-assemble into diverse complex structures made from their monomeric units under the control of enamelysin protease [23–25] to form spherical subunits, which may afterwards serve as crystal nuclei [26–28]. The foremost amorphous calcium phosphates (ACP) [29] settle at the border of the enamel and dentine in the form of thin ribbons 10-15 nm in length and 1-2 nm in thickness [30, 31]. The study by Iijima [32] shows that amelogenin is absorbed on these mineral ribbons suppressing their growth in thickness. Thus, there is the appositional growth of ACP preferably in the direction perpendicular to the tooth surface [18, 33], the amelogenin-based supramolecular assemblies being the key agent responsible for the origination and directionality of CaP growth [19] probably in combination with corresponding effects of ameloblastin [25]. During the later secretory stage of enamel calcification the matrix proteins are specifically broken down with enamelysin (MMP-20) allowing the controlled transformation of the ACP phase into the final crystalline apatite [29, 34].

During the maturation stage, the partially split matrix proteins are almost completely removed by KLK4 protease providing the space yielded by the lost proteins for CaP crystal growth [34]. Robinson [35] stresses that the removal of matrix protein seems to be a prerequisite for crystal growth, presumably providing mineral ions with access to the growing crystals, and argues that the initial removal of proteins in interior, i.e., first formed, enamel results in maturation growth accelerating from inside to outside. In general, it can be concluded that the molecular events of enamel formation during the secretory stage have been elucidated to considerable extent, and recent detailed information is also available on the molecular mechanisms of enamel maturation [17], though obviously lesser attention has been paid to the crystallographic effects of particular processes composing the molecular machinery of enamel maturation [31].

In any case, however, much less is known about how the particular processes contributing to enamel formation are orchestrated to produce the complex patterns of enamel microarchitecture characterizing particular clades of mammals, and how the distribution of particular enamel patterns over the particular loci of a tooth is controlled. Detailed comparative information on developmental mechanisms modifying the enamel patterns of particular mammalian clades is largely missing. The questions of which of them present true synapomorphies of all mammals and which were stepping stones in the evolution of mammalian amelogenesis [36] remain thus largely unanswered.

Detailed developmental information is available for a few model taxa, mainly for mouse, rat and human. Unfortunately, in dental respects, including enamel microarchitecture, these taxa—muroid rodents and anthropoid primates—are extremely derived, showing a large set of phylogenetic rearrangements from the basic mammalian dental type. It can be expected that this might also concern some of the amelogenetic processes observed in them. Information on enamel formation in taxa retaining the ancestral form of mammalian dentition is urgently required. First of all, this refers to those taxa bearing the tribosphenic molar, the ancestral form of molar teeth and a key apomorphy of mammalian dentition [37–40]. Tribosphenic molars are characterized by a regular radial arrangement of enamel prisms (PE), continuous from the enamel-dentine junction (EDJ) to the occlusal surface, with an interprismatic matrix (IPM) of disorganized CaP crystallites in between the prisms and a thin surface cover of aprismatic enamel, APE [13, 14, 41–43]. Developmental information available on the amelogenesis of tribosphenic teeth suggests that, from the beginning of amelogenesis during embryonic development, exclusively prismatic enamel is produced, while IPM and APE first appear only shortly prior to tooth eruption [44]. Until then the enamel coat composed exclusively of radial prisms is unconsolidated and imposes no mechanical constraints on the enlargement of the tooth and the shaping movements of the dental papilla. In this respect, radial prismatic enamel plays the role of structural scaffold allowing the enamel coat of mammalian teeth rapidly to attain a considerable thickness and retain it notwithstanding further shaping movements of the tooth primordium. Delayed enamel maturation (including the late appearance of IPM and APE) then prolongs the time available for tooth enlargement up to tooth eruption. It was suggested that the combination of these processes is the essential novelty of the mammalian amelogenetic dynamic and possibly became a key driving factor of mammalian dental evolution [45]. Unfortunately, which processes underlie these innovations, in terms of mineralization dynamics and crystal growth, and how their variations effect the final functional and biomechanical qualities of the adult tooth crown is largely unknown.

**Fig 1:**
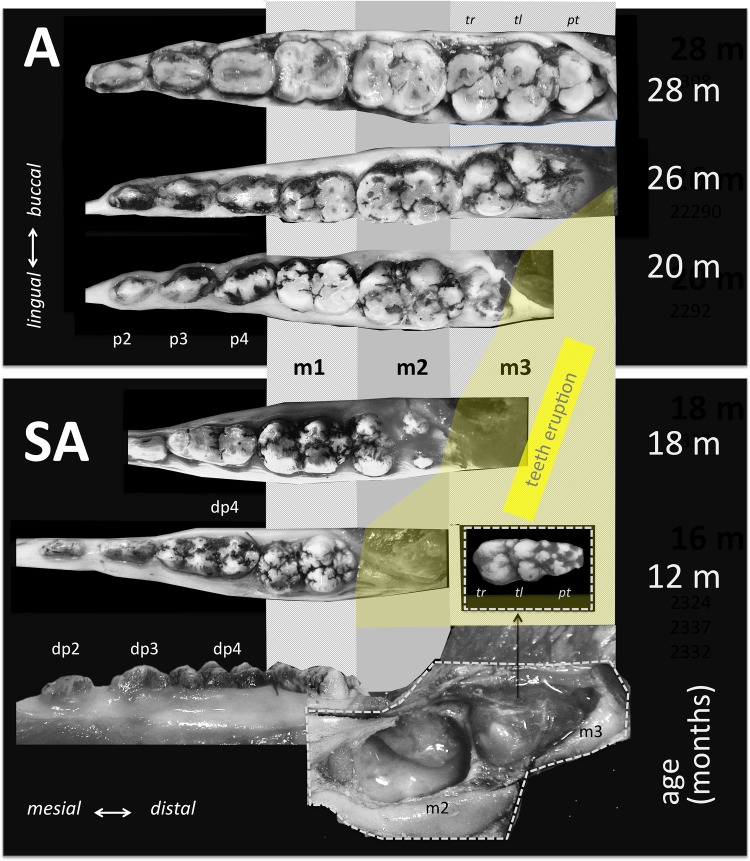
Mandibular dentition and molar eruption in subadult (SA) and adult (A) minipig representing the age span covered in this study. Note the embryonic stage of m3 development (entirely covered by a vascularized dental sac) in subadult individuals.

**Fig 2:**
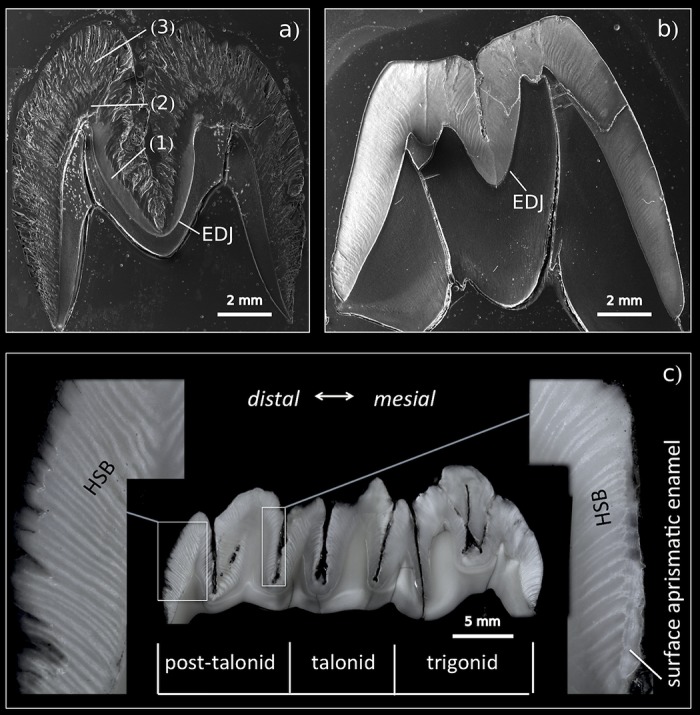
Cross-section of the mesial part (protoconid-metaconid) of m3 (SEM) in (a) an immature (17-month-old) and (b) mature (30-month-old) minipig. The immature tooth illustrates the heterotopy of enamel maturation: the simultaneous appearance of mature compact enamel close to the EDJ (1), a partly mature enamel segment (2), and outer immature enamel (3); EDJ = enamel-dentine junction. (c) longitudinal section of semimature m3 (20-month-old) individual with a macroscopic view of Hunter-Schreger bands (HSB) and outer aprismatic enamel.

To examine aspects of these processes in detail, we analyzed the crystallographic characteristics of enamel at different stages of pre-eruptional development using the lower distal molar (m3) of the miniature laboratory pig as a model (for basic odontologenetic characteristics see [46–48]—(Fig 1). Although the bunodont molar teeth of pigs retain only little of the original tribosphenic design, they share many of its enamel characteristics. In contrast to the derived enamel microarchitecture in the most frequent model taxa (mouse, rat, human), pig teeth exhibit essentially a uniform radial prism arrangement, with regularly dispersed IPM and APE (Fig 2). The tooth we studied (m3) is the largest tooth of adult dentition, responding to extreme functional demands with a very thick enamel. In contrast to rodent models, the tooth development is particularly prolonged (calcification begins at the age of 8 months, eruption is completed at 23 months), which provides a chance to trace the diverse steps of enamel formation and enamel maturation in greater detail. We believe that this is the most suitable model for revealing the specific roles of developmental processes and for distinguishing putative heterochronies and heterotopies among them at a high scale of resolution. In addition, a very thick enamel coat makes it possible to collect the large CaP samples required for crystallographic studies and, hence, to compare the states of particular crystallographic variables in different parts of the tooth crown as well as to analyze the patterns of spatial variation in the development of CaP crystallites in different enamel coat layers.

The standard crystallographic technique applied in this study was X-ray powder diffraction. It provides information about atomic positions within the crystal structure. The qualitative and structural properties of the studied materials are indicated by the diffracting peak position on the 2*θ* scale and its intensity. Yet, small crystal dimensions and lattice imperfections (preferentially dislocations) result in a broadening of the peak shape. These dislocations have the effect of dividing the original crystals into much smaller domains. These domains scatter incoherently with respect to one another and thus behave like very small crystals; they are usually described as crystallites. Finally, the extent of the dislocations that cause the subdivision of the original larger crystal into domains is referred to as microstrain [49].

## Materials and methods

A series of enamel samples were collected from 18 miniature laboratory pigs aged between 16 and 30 months (for a detailed description see S1 Table) provided by the Institute of Animal Physiology and Genetics in Liběchov, Czech Republic. We were allowed to extract jaws from the individuals sacrificed on diverse terms in 2013 and 2014 for the purposes of several research projects conducted in the Institute (comp. e.g. [50–52] etc.). The pigs were euthanatized by gunshots using a forehead strike by captive bolt pistol (comp. AVMA Guidelines for the Euthanasia of Animals). Transgenic minipigs have been kept in the Institute for more than 40 years under highly standardized conditions responding to current Czech regulations and guidelines for animal welfare and with approval from the State Veterinary Administration of the Czech Republic. We ensured that all components of the respective projects including all particular procedures were carried out in accordance with the Projects of Experiment approved by the Animal Care and Use Committee of the IAPG AS CR, v.v.i. (Libechov, Czech Republic), following the rules of the European Convention for the Care and Use of Laboratory Animals and related Czech regulations. We used female individuals of the same breed, all kept under highly standardized conditions. The project of this study (by IH) was approved by the Animal Care and Use Committee of the Faculty of Science, Charles University, Prague. The third molars (m3) of each individual were extracted from the right mandible to obtain samples for X-ray powder diffraction (XRD) experiments and from the left mandible to obtain samples for imaging using scanning electron microscopy (SEM) and microindentation testing (MiT) (for a list of variables see S2 Table).

The samples for XRD investigation were manually disintegrated into fragments of potentially pure enamel from the selected tooth areas (S1 Fig) according to [53]. The inner/outer enamel parts were taken in the area of the talonid. To confirm the purity of enamel fragments an optical microscope and scanning electron microscope (Tescan Vega3 XMU) were used. Finally, the pure enamel was ground under acetone in a CoorsTek alumina mortar.

We performed X-ray powder diffraction measurements using a Bruker D8 Discover diffractometer equipped with a linear LynxEye detector and a germanium primary monochromator providing CuK_*α*1_ radiation (= 1.54056 Å). Data were collected in the 2*θ* range of 5—122°with a step size of 0.013°, a counting time of 7 seconds at each step, and a detector angular opening of 3.7°. The analysis of diffraction line broadening was accomplished using Le Bail whole-pattern fitting implemented in the Full-Prof software program [54]. The standard reference material LaB_6_ SRM 660b (NIST) was used to characterize the instrumental resolution function. The agreement factors of the Le Bail fit mostly clustered around 1.6 (*χ*^2^).

SEM/MiT tooth samples were cut vertically into halves in the bucco-lingual direction (at the talonid area). These tooth halves were subsequently embedded in epoxy resin and polished. We also used the remaining tooth halves, gently detaching the surface layer of the vertical cut (talonid area) in a way that followed the natural cleavage planes, to uncover the actual enamel texture. All samples were first documented by means of digital photography (in the optical microscope); then, micromechanical properties were analyzed (samples embedded in epoxy); and, finally, SEM images were taken. We first took images of all polished SEM samples (gold sputtered); then the gold was removed and the samples etched by 3% HCl (3 s) and subsequently gold sputtered again. SEM images were taken in the secondary electron (SE) mode, in high vacuum at 20-30kV and at working distances of 7-12 mm using a Tescan Vega3 XMU.

**Fig 3:**
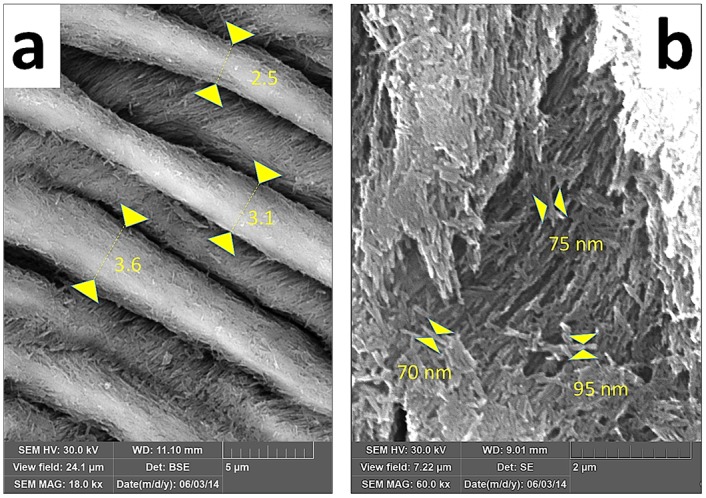
Techniques of measuring prism width (a) and thickness of crystallite aggregates (b).

The width of enamel prisms and the thickness of crystallite aggregates were analyzed from SEM images using ImageJ and IC Measure software for on-screen measurements (Fig 3).

Micromechanical properties were characterized by an instrumented microindentation hardness tester (Micro-Combi Tester; CSM Instruments, Switzerland). For each specimen, at least 10 indentations were performed per each cut surface and selected location. In each selected location, the indents were made in a line parallel with the outer edge of the tooth section, i.e. at the same distance from the tooth surface. The indentations were performed with a Vickers indenter (diamond square pyramid, angle between two non-adjacent faces 136°); details of the experimental geometry are described elsewhere [55]. The indenter was forced against the cut surfaces with the following parameters: load 0.4905 N (50 gf), load time 6 s, and linear loading/unloading rate 0.417 N/s (25,000 mN/min). For the given materials and experimental setup, the size of the indents on the cut surface was well above 10 *μ*m. Consequently, the results should not be overly sensitive to micro- and nanometer scale inhomogeneities in the enamel structure. Curves showing applied force (*F*) vs. penetration depth (*h*) were used to calculate indentation hardness (*H*_IT_), indentation modulus (*E*_IT_), indentation creep (*C*_IT_), and the elastic part of the indentation work (*η*_IT_). *H*_IT_ and *E*_IT_ are the most common properties of microindentation testing, which, in our study, were determined according to the well-established theory of Oliver and Pharr [56]. Indentation creep, *C*_IT_, gives the resistance of a material to a long-term load. The higher the value of *C*_IT_, the lower the resistance to a long-term load. Microindentation creep was calculated as the relative increase in the penetration depth during full load: *C*_IT_ = (*h*_2_ − *h*_1_)/*h*_1_ × 100%, where *h*_1_ is the penetration depth of the indenter into the specimen at the beginning of the full load and *h*_2_ is the penetration depth at the end of the full load. The elastic part of the indentation work (*η*_IT_) is related to the elasticity of the material. Completely elastic material, such as elastic rubber, would have *η*_IT_ = 100% and would completely return to its original shape after unloading, i.e. there would be no permanent imprint of the indenter on the material surface. The elastic part of the indentation work was calculated on a relative scale as: *η*_IT_ = *W*_elast_/*W*_total_ × 100%, where *W*_elast_ is the elastic indentation work (the area between the loading and unloading curves) and *W*_total_ is the total indentation work (the total area under the loading curve). Detailed descriptions of the calculation of all four quantities determined from microindentation experiments (*H*_IT_, *E*_IT_, *C*_IT_, and *η*_IT_) are given in the manual of the Indentation 5.18 software [57].

Statistical analyses were performed in Microsoft Excel and STATISTICA 8.0 software. The Gauss error function *f*(*x*) = 0.5*a* ∗ (1 + *erf*(*b* ∗ (*x* + *c*))), linear regression *f*(*x*) = (*x* ∗ *a*) + *b* and polynomial regression *f*(*x*) = (*a* ∗ *x*^2^) + (*b* ∗ *x*) + *const*., where a, b and c are refined parameters, were fitted to the observed data using gnuplot [version 4.6.4-2].

## Results

All stages of m3 development covered by our study (including 16- to 18-month-old individuals with embryonal teeth that had not yet attained their final size and shape—see (Figs 1SA and 2) exhibited a roughly identical enamel cover thickness corresponding to that of the adult m3. Yet, particular stages differed markedly both in crystallographic variables and enamel histology. A complete survey of the measured values is available in S2 Table. Except for microstrain and *C*_IT_, all crystallographic variables were significantly age-dependent and most of them showed clear mutual correlations (S4 and S5 Tables; S2 and S4 Figs).

**Fig 4:**
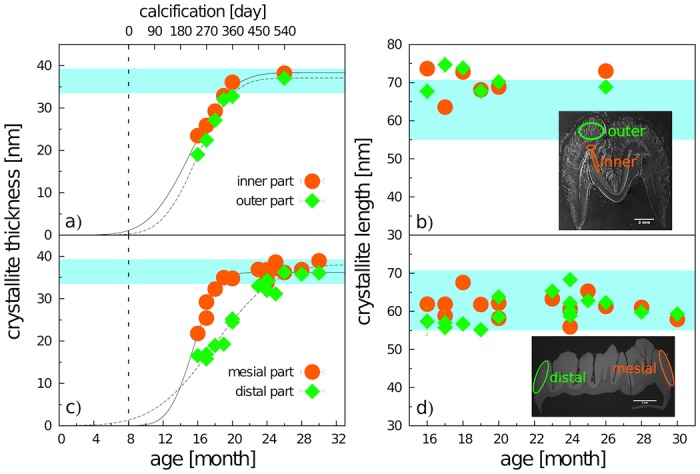
Crystallite thickness (a,c) and crystallite length (b,d) revealed by X-ray diffraction of the inner and outer enamel (a,b), and the mesial and distal enamel (c,d) plotted against the postnatal age of particular individuals (abscissa, lower) and day of m3 calcification (abscissa, upper)—mean values of particular individuals with SD error bars (mostly covered by the marks). The blue band indicates the range of mature enamel crystallite size estimated both from our previous observations and from data by Daculsi and Kerebel [31], Daculsi et al. [58], and Simmer and Fincham [8]. The beginning of calcification (the zero point of the upper abscissa scale) is taken from Tonge and McCance [47] and Wang et al. [48]. The ovals indicate sampling areas for either inner and outer enamel and the mesial or distal tooth part, respectively.

The crystallite size in the adult m3 varied between 33.8 and 38.9 nm in thickness (**l**_**a**_ crystallographic direction), and 56 and 70 nm in length (**l**_**c**_ crystallographic direction). We found no significant difference between adult and subadult m3 in the length of crystallites (Fig 4b and 4d). In contrast, the thickness of crystallites in individuals younger than 21 months was significantly smaller than in adults, and exhibited a clear age dependency fitted smoothly with a Gauss error function (Fig 4a and 4c), conforming to a model of gradual growth with a mean monthly increment of about 3.15/3.44 nm (11%; 14.5%) at the inner/outer zone of enamel coat. The zero point of the error function suggests that the growth of crystallite thickness begins at a postnatal age of about 5-6 months for the inner zone and 7-8 months for the outer zone. In the inner zone, the adult crystallite size was achieved at around 20 months—ca. 390 days of calcification (DoC). The slopes of the error function for crystallite thickness in the mesial and distal part of the tooth differed significantly, suggesting an average monthly increment of 4.4 nm (16%) at the mesial and 3.8 nm (15%) at the distal part (Fig 4a). Compared to the mesial part, the zone of adult values was achieved with about 5 months delay in the distal part (20 vs. 25 months of postnatal age).

**Fig 5:**
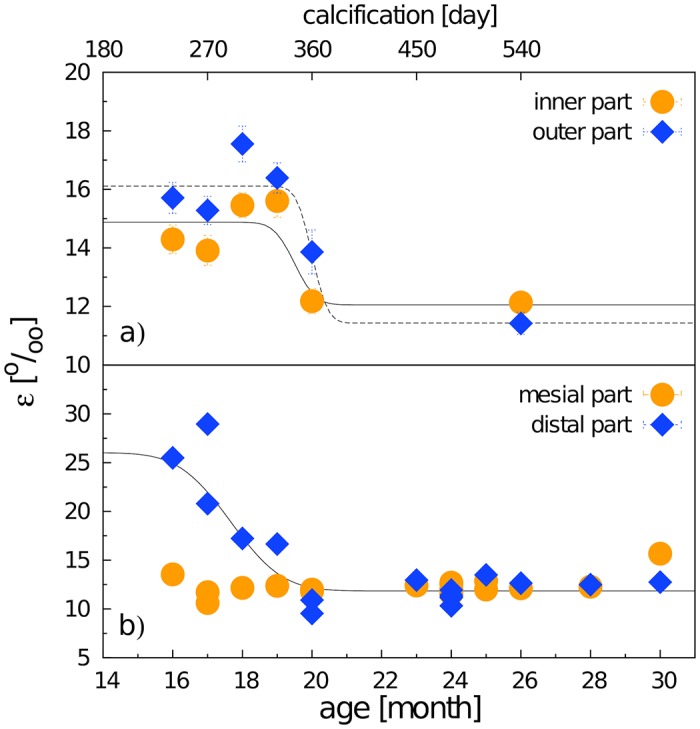
Variation of microstrain during the maturation stage of calcification in (a) the inner and outer enamel parts, (b) the mesial and distal tooth parts. Mean values of particular individuals with SD error bars (mostly covered by the marks).

Microstrain computed for the mesial part of the crown showed consistent values of around 12.35‰ in all individuals, a value which was attained at the distal crown part only at the age of 20 months after a dramatic drop from much higher values in younger individuals (Fig 5b). The difference between 16- and 20-month-old individuals was more than 50%. From the age of 20 months, the microstrain values showed no significant variation. Prior to that age, the microstrain values of outer enamel were invariantly higher than those of inner enamel. Yet, for both the inner and outer enamel layers, microstrain in immature individuals (<20 months) was higher than that in older individuals, showing a steep transition within the month prior to the age of 20 months (Fig 5a).

**Fig 6:**
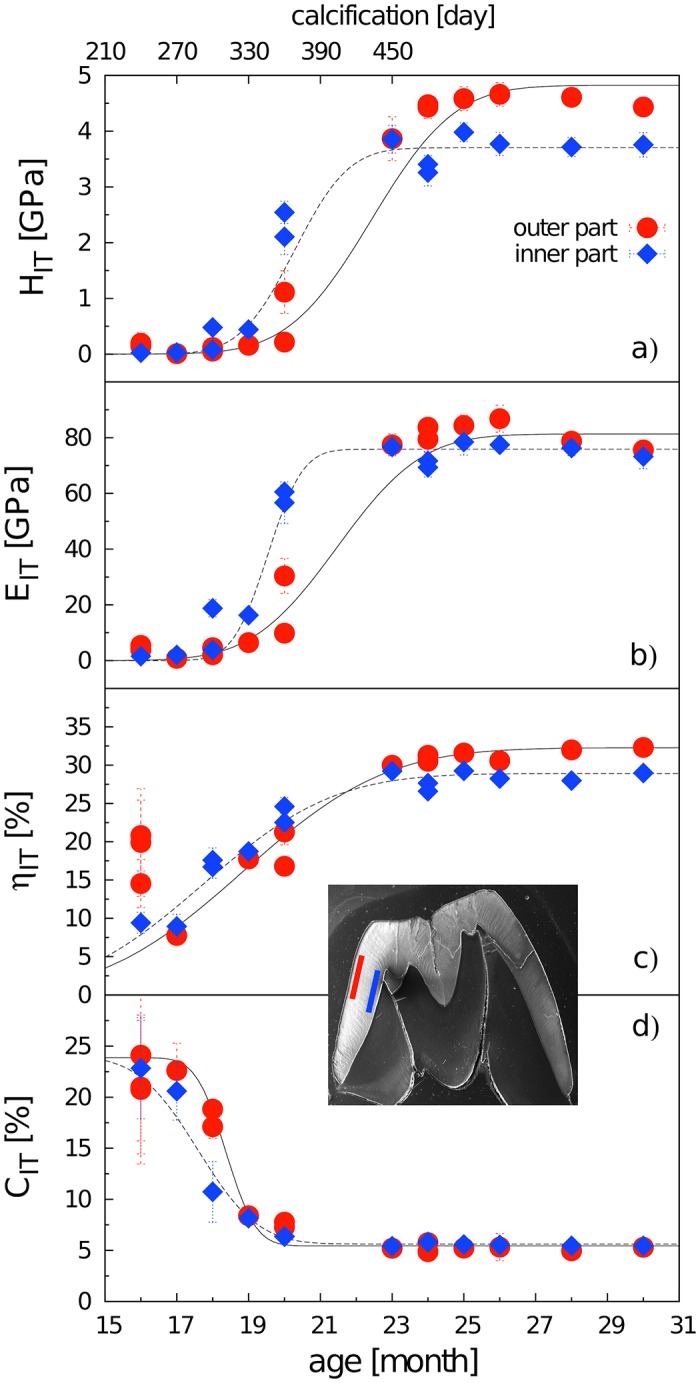
Mechanical parameters (a) *H*_IT_, (b) *E*_IT_, (c) *η*_IT_, and (d) *C*_IT_ and their relationships to enamel formation. Each point represents an average value calculated from 10 microindentation points. For d) the error function parameter a from the inner enamel part was set to the value of the same parameter for the outer enamel to prevent instability in the fit. Mean values of particular individuals with SD error bars (mostly covered by the signs).

Data concerning the mechanical properties of enamel revealed by indentation analyses are summarized in Fig 6. Despite certain differences among particular variables, they all show an abrupt shift within the period between 18-23 months of animal age (i.e. 300-450 DoC): the hardness *H*_IT_, stiffness *E*_IT_, and elasticity *η*_IT_ of enamel increased, while the behavior of the indentation creep parameter *C*_IT_ was quite the opposite, showing that during the late stage of maturation the enamel essentially increases its stiffness, hardness, elasticity, while its capacity for plastic deformation dramatically decreases. Until the postnatal age of 20 months, the inner and outer enamel layers exhibited roughly the same values (close to zero) of *H*_IT_ and *E*_IT_, yet, adult teeth (>23 months) *H*_IT_ values for the outer enamel zone were significantly higher than those for the inner zone (Fig 6a), and the transition to adult values for both variables was clearly steeper in the inner enamel. From the age of 23 months the enamel mechanical properties showed almost no variation.

In short: we demonstrate a gradual fluent growth in crystallite size, continuous throughout the pre-eruptional period, combined with an abrupt decrease in enamel microstrain and a rapid switch in enamel mechanical properties between 18-23 months. The inner/mesial enamel part is about 3-5 months ahead in embryonic development compared to outer/ distal parts. Full maturity of all loci of the minipig m3 is achieved around 25—26 months of age (i.e. the whole calcification process takes around 510—540 days).

**Fig 7:**
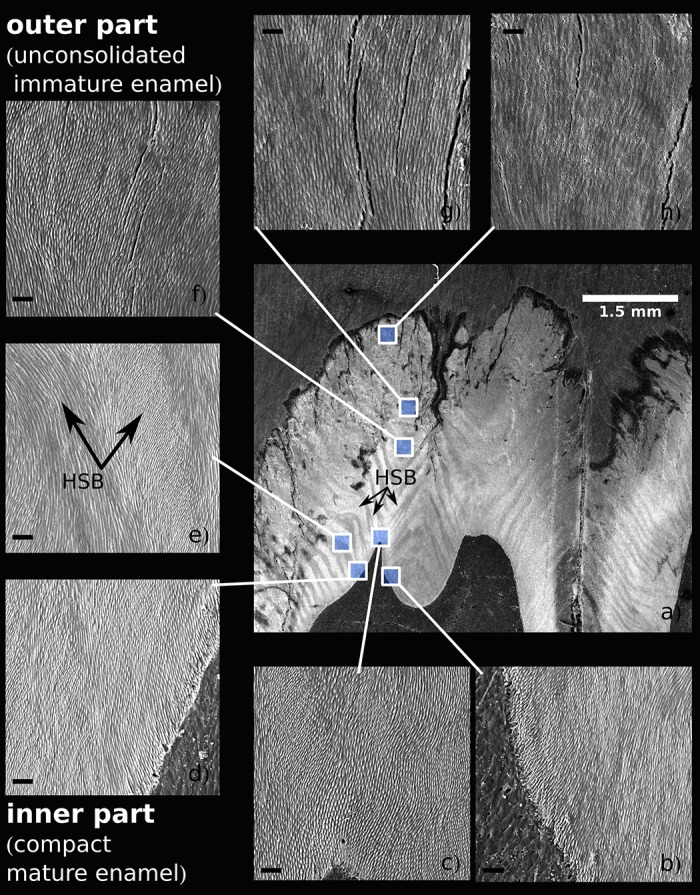
A cross-section of the mesial part of m3 in a 17-month-old individual (a) and the structure of the enamel at different parts of the enamel coat (b-h), all at the same magnification, SEM, acid etched, scale bar 10 *μ*m. Note the apparent differences in enamel texture and prism width between the mature inner enamel (b-e) and the immature outer enamel (f-h). HSB—Hunter-Schregers bands.

The SEM images of the completely mature enamel show a compactly packed radial prismatic pattern with a dense interprismatic matrix (IPM) uniformly dispersed across the overall enamel area. The radial organization of the enamel coat is further accompanied by distinct macroscopically visible radial strips of multiserial Hunter-Schreger bands (HSB) (Figs 2c and 7). In contrast to the radial prisms continuous from EDJ to the crown surface zone, the condensed tangentially oriented prisms constituting the HSB (Fig 7e) are restricted to a particular HSB strip, a portion of them obviously derived from neighboring radial prisms. The surface of the crown is covered by a distinct layer of aprismatic enamel (Fig 2c). All these characters of adult enamel were completed first at the mesial part of the tooth in 20-month-old individuals, i.e. at the age when the eruption of m3 began with a noticeable bulging of the gingiva. The paraconid, the first cusp appearing above the gingiva, was completely erupted at 23-24 months. Even at that time, when the mesial (trigonid) tooth part was already fully developed, the enamel structure of the distal part of the tooth retained its embryonic characters with incomplete maturation (Fig 7).

**Fig 8:**
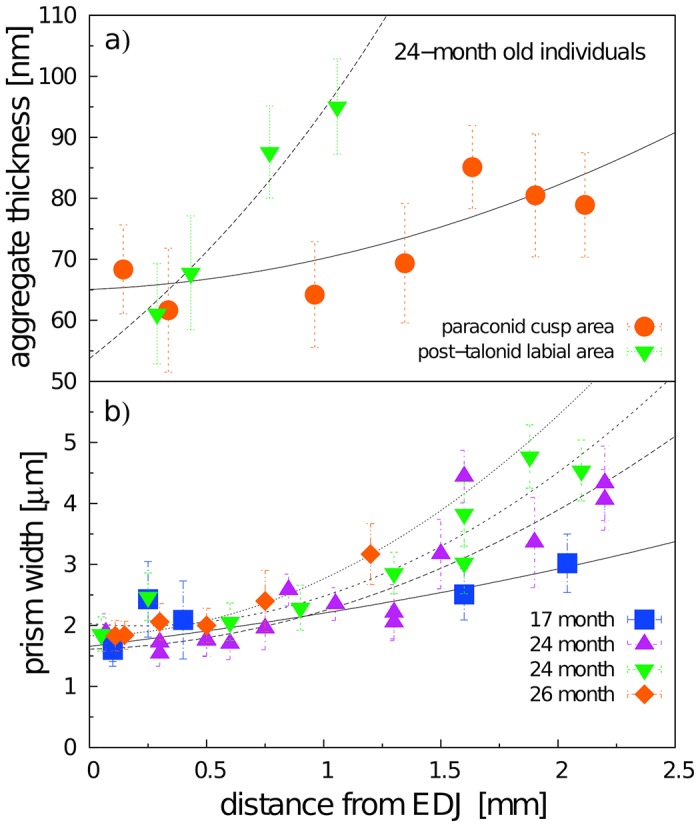
Mean width of radial prisms (a) and mean thickness of core crystals (b) at particular distances from the EDJ measured from SEM images (2000x magnification). Each point represents an average value calculated from at least 30 measurements, in some cases up to 240 measurements, with SD error bars.

The width of the radial prisms varied within the range 1.5—4.8 *μ*m with a gradual increase from EDJ to the crown surface both at the mesial and distal parts of the tooth. The respective trend was present both in immature and mature enamel (Fig 8b), yet in the outer zone the prism width of immature enamel (1.8-3.2 *μ*m in 17 month old individual) was significantly smaller than in 24 month old individuals (2.6-5.3 *μ*m). Corresponding trends were observed also with the mean thickness of the core crystals of the radial prisms, elementary structural units visible in the SEM images (Fig 8a). The mean thickness of these crystals in the inner zone (close to EDJ) was nearly the same both in the mesial and distal parts of the tooth (64 vs. 63 nm) while at the outer enamel the respective values in the distal part (94 nm) were significantly higher that in the mesial part (75 nm).

**Fig 9:**
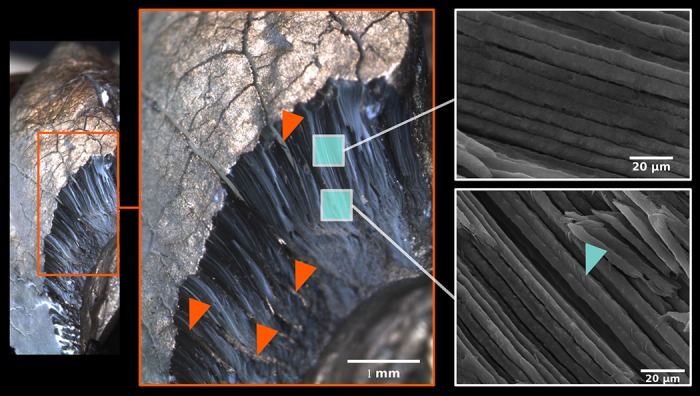
Optical macrophotography of the post-talonid part of the m3 crown in a 20-month-old individual. Note the dense arrangement of prisms, the absence of IPM and APE, and the structural fissures among compact blocks of prisms, partly infilled by a non/mineralized compound (red arrowheads). Natural surface, no polishing, no acid etching, gold coating. Blue arrowhead: isolated IPM aggregates at inner part of enamel prisms.

**Fig 10:**
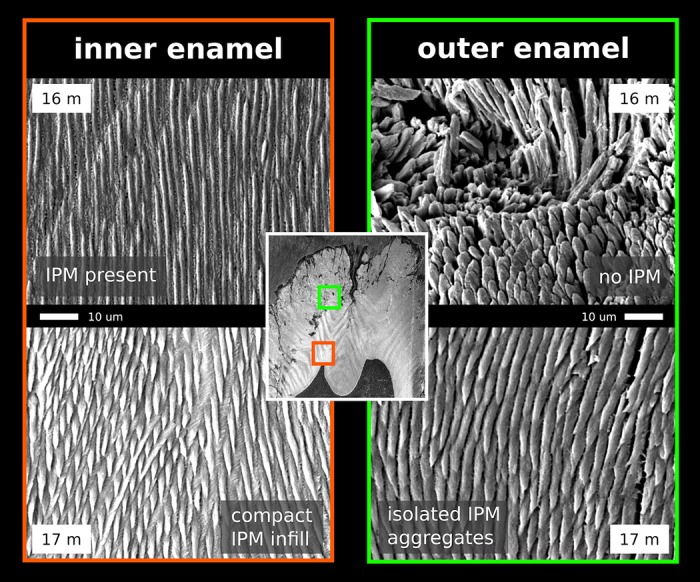
Enamel microstructure at identical loci of the mesial part of m3 in 16- and 17-month-old individuals (SEM, no acid etching). Note the complete development of dense IPM at the inner part of the crown while only the first signs of IPM appear at the outer part.

The embryonic enamel of the youngest individuals of our series (age 16-17 months) was restricted to long fibers of radial enamel rods (covered by well-defined sheets) showing a considerable spatial divergence toward the crown surface (Fig 9). Only a narrow deep zone along the EDJ with the dense packing of enamel rods exhibited the regular appearance of adult-like IPM (Figs 7 and 10). The prisms of the outer zone showed an erratic termination lacking pronounced spatial integration, with numerous anastomosing divergences and a large amount of empty spaces without mineralized infill, in particular in the distal part of the tooth. The rough crown surface (after removal of the remnants of the enamel organ) showed only loose prisms irregularly interconnected by anastomosing fibers (Fig 9). The aprismatic surface coating, characterizing adult mature enamel, was completely absent in these stages (S3 Fig). The deep layer of mature-like enamel along the EDJ and the outer zone of the loose anastomosing rods were separated by a distinct transitional zone exhibiting a regular spatial arrangement of prisms and the incomplete development of IPM. In the trigonid of 16-month-old individuals, the transitional zone was quite narrow; in 17-month-old individuals it amounted to ca. 1/5, and in 18-month-old individuals it covered about a half of the enamel thickness.

The very thick layer of loose prisms of embryonic enamel was split into irregular spatial blocks (supposedly due to the continuous growth of tooth size during the pre-eruption period) separated by deep fissures initially lacking any mineralized infill. CaP mineralization of these between-block spaces began from the EDJ. In the deeper part of the enamel coat it took the form of prismatic crystallization producing the HSB pattern (Fig 9). In the outer zone the HSB pattern was less distinct and the fissures were finally covered by the surface APE with well-marked perikymata along deeper cervical parts of the crown.

**Fig 11:**
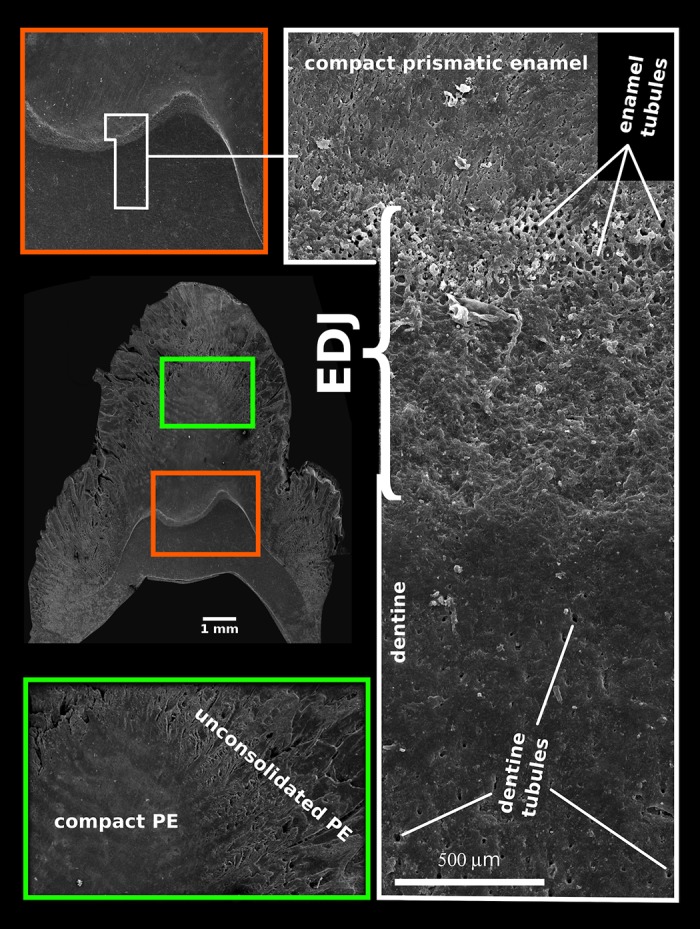
SEM images of selected tooth parts of a 17-month-old individual showing the transition zone between dentine and enamel (EDJ) and similarities between the shape and size of the dentine and enamel tubules (acid etched).

All the above-mentioned changes associated with enamel maturation began in the zone of the EDJ. Fig 11 shows that in embryonic teeth the EDJ represents a thick zone, morphologically distinct and histologically much different from the EDJ of adult teeth. Its enamel face is characterized by numerous enamel tubules with strongly mineralized tubular walls that are equal in diameter both to dentine tubules and the bases of densely packed enamel prisms appearing in their continuation.

## Discussion

The term of enamel maturation specifically denotes the stage when the final thickness of the enamel layer is already achieved and amelogenetic processes are predominantly oriented to the removal of water and organic materials, mineralization, and promoting enamel mechanical properties [35]. When the enamel matrix is first laid down, it is 80-90% protein and fluid and only 10-20% mineral by volume, while the mature enamel is >90% mineral by volume [59]. In fact, maturation is an enormously complicated process during which the products of the secretory phase, both structural and material, integrate to form the adult enamel coat with all the fine details of its microarchitecture and all its exceptional mechanical properties [6, 17, 19].

During the maturation stage, ameloblasts lose their Tomes processes and their secretory activity takes the form of a mass pumping of calcium ions into the space of maturating enamel, together with some calcium-binding proteins that do not appear during the secretory stage [6]. This causes fluctuations in the pH of the enamel fluids [60], which among other things prevents enamel demineralization and provides pH conditions optimized for the functioning of matrix degrading enzymes, mainly KLK4 produced specifically during this stage [19]. A recent review of this topic stresses that the maturation stage is the least-well-known part of odontogenetic processes, and that only now are we beginning to understand the principles of enamel biomineralization, its molecular control mechanisms and their possible roles [17].

In this paper, we present the results of a crystallographic study of enamel maturation using a minipig m3 as a model. While focusing on sampling techniques designed to prevent methodological bias in the estimations of crystallographic variables [53], we found several consistent trends characterizing pre-eruptional enamel maturation, which can be summarized as follows. (1) The size of crystallite exhibits a fluent gradual enlargement that terminates with the attainment of adult values at 20 months of postnatal age (i.e. the stage when the eruption of the mesial part of the tooth begins). (2) This size enlargement concerns crystallite thickness not crystallite length, for which adult values were clearly achieved prior to the period covered by our study (16-31 months of postnatal age). (3) There is well-marked structural heterotopy in the course of these processes: the maturation of the outer enamel layer and the distal part of a tooth are considerably delayed in comparison to that in the inner enamel layer and the mesial part of the tooth. In contrast to the gradual manner of crystallite size enlargement, (4) the microstrain—a measure of crystallographic imperfections—shows a different dynamics: an abrupt decrease over a short period prior to the beginning of tooth eruption, synchronous with (5) an abrupt switch in the state of the quantitative variables characterizing the mechanical properties of the enamel, particularly the overall hardening of the enamel, which attains adult values immediately prior to eruption. (6) Until then, the mineralized enamel (as visualized by SEM images) is organized in the form of parallel radial prisms with well-developed sheets, but not integrated by a crystallized interprismatic matrix. (7) The mass crystallization of IPM (revealed by SEM imaging) and finally the appearance of a surface layer of aprismatic enamel is synchronous with maturation processes (4) and (5). (8) The IPM infill of deeper cracks separating the compact blocks of radial prisms takes the form of macroscopically visible columns of densely compressed tangential enamel rods, constituting conspicuous Hunter-Schreger bands characteristic of porcine adult molars (Fig 2c). (9) Both in embryonic and adult enamel, the width of radial prisms increases from the EDJ to the crown surface, similarly to the mean size of the core crystals composing them. (10) At the EDJ, where the spatial arrangement of prisms is quite compact, and even in the earliest embryonic tooth of our series, the prisms (or prismatic sheets) seem to grow from the enamel tubules that are clearly directly continuing the dentine tubules.

In regards to general aspects of mammalian enamel maturation, our results deserve some further comment, particularly with respect to differences from the standard mouse model of enamel maturation. (i) First, we should stress the difference in size of the core structural units visualized by SEM imaging and of the elementary CaP crystallites revealed with the aid of X-ray powder diffraction. It suggests that in porcine enamel, in contrast to enamel of the mouse incisor but similarly as in enamel of tribosphenic molars [44], the core crystals are actually composite aggregates of the elementary crystallites. The thickness of the crystals—i.e. the core aggregates, the smallest structural units distinguishable in the SEM images—varied from 51 to 105 nm (showing an increasing trend from the EDJ to the crown surface, with mean values of 65 to 85 nm). Considering the mean value of adult crystallite thickness (33-38 nm) this suggests that the core crystals are formed by two to three elementary crystallites. In terms of SEM imaging, the appearance of distinct crystals (aggregated from several elementary crystallites) seems to be a pertinent characteristic of the mature enamel. This takes place at the time of an abrupt decrease in microstrain, which raises the question of the degree to which crystallite aggregation plays a role in the mechanism compensating lattice disorders of individual crystallites formed prior to that stage. In any case, as the size of aggregates shows no influence upon the state of the crystallographic size variables of enamel CaP, it appears that, contrary to the proposal of Simmer et al. [18], the aggregation of crystallites is not the driving force behind enamel mineralization.

(ii) The comparison of particular ontogenetic stages undertaken in this study demonstrated a fluent gradual increase in the thickness of individual crystallites composing the prisms (with a monthly increment of ca 3.8 nm). This suggests that the continuous absorption of calcium phosphate onto the lateral faces of nuclear CaP crystallites is the key factor in their maturation. Such a view is consistent with numerous studies on the embryonic growth of CaP crystallites employing high-resolution transmission electron microscopy [8, 31, 58, 61], including their conclusions that the gradual increase in crystallite thickness is preceded by a rapid elongation of crystallite length prior to the beginning of the maturation stage proper.

(iii) The abrupt decrease in microstrain observed during the last stages of the maturation process (more than 50% compared to that in 120 DoC) is, to our knowledge, reported here for the first time. Microstrain values can serve as a quantitative measure of lattice defects in the crystal structure, which can induce significant variations in its physical and mechanical properties [49]. A high amount of several types of crystal defects (such as edge and screw dislocations) in fetal enamel was reported by Cuisinier et al. [62]. Špoutil et al. [44] suggested that high values of microstrain found in the tribosphenic molars of bats may arise due to the amelogenetic dynamics characterizing that tooth type (simple radial enamel, delayed maturation). Robinson et al. [59] surveyed sources of crystallographic imperfection in enamel CaP in detail, noting that planar defects parallel to the long c-axis of the crystallite are particularly common there. Terminal hydrolysis, epitaxial overgrowth, and screw dislocations propagated in the direction of the c-axis were all proposed to explain the source of this enamel CaP lattice disorder [59]. The abrupt decrease in the amount of lattice defects that we observed at the terminal maturation stage can be ascribed either to the healing effect of the mutual aggregation of elementary prismatic crystallites and/or to a change in the environment of the crystallization events which take place within that stage, namely the appearance of IPM. It seems quite possible that the hyperalkalic conditions driven by the active disappearance of water and residual proteins may promote a considerable reduction in lattice disorder in IPM crystallites, yet for obvious reasons it was beyond the scope of the method to support this view with instrumental data. (iv) It is also worth mentioning the fact that the dramatic shift in enamel micromechanical properties revealed by microindentation analyses appeared just within the terminal maturation stage. In contrast to microstrain, which showed no significant correlation to any of the crystallographic variables (including the size of crystallites), three out of four micromechanical variables (hardness, stiffness, elasticity) were mutually strongly correlated at a high level of statistical significance (S5 Table). Surprisingly, they showed no significant correlation with crystallite thickness, at least in the inner enamel. The fourth micromechanical variable, *C*_IT_, differed substantially: for the inner enamel it exhibited no significant correlation with any other variable except for the *C*_IT_ of the outer enamel. This variable, which quantifies the capacity to respond to long-term load, exhibited a rapid decline between postnatal months 17 and 18. This preceded the changes in other variables, including the *C*_IT_ of the outer enamel, which correlated significantly with other micromechanical variables (S5 Table). This means that until the rapid drop in *C*_IT_ shortly prior to tooth eruption the enamel coat is not capable of resisting the permanent deformation and thus presents no essential constraint to the enlargement of tooth size generated by the 3D growth of the dental papilla (in our porcine model, this was apparent, for example from the smaller sizes of developing teeth compared to those in adult individuals comp. Figs 1 and 2). Similarly as in tribosphenic molars, the structural organization of immature enamel, which is composed exclusively of loose radial prisms, seems to be a key prerequisite for perieruptional tooth enlargement [45]. The reshaping capacity is then terminated by the appearance of IPM, spatially integrating individual prisms, and the surface cover of aprismatic enamel. All these results suggest that the mass production of IPM is the proper agent of the final stage of enamel maturation, by which the exceptional mechanical properties of adult enamel are created.

(v) Consequently, in terms of crystallization dynamics, enamel formation seems to include two (or three) distinct processes: (a) the early appearance of a radial prismatic scaffold preforming the total thickness of the enamel coat, which simultaneously allows the further enlargement and reshaping of the immature tooth, and (b) delayed mineralization of the interprismatic matrix postponed to the late stage of maturation, which terminates with (c) the appearance of surface aprismatic enamel prior to the eruption of the finally shaped tooth crown. The respective processes seem to differ not only in their timing and functional effects, but, as mentioned above, also in the dynamics of crystallite growth and presumably the amount of lattice disorder. The heterochrony of these processes has been proposed as an essential factor in the developmental dynamics of the tribosphenic molar, a key apomorphy of mammalian dentition [45]. With the aid of crystallographic analysis we identified the same pattern also in the derived molar type of a pig. The sharing of the same patterns in phylogenetically quite distant taxa indicates that the observed organization of enamel maturation may represent a common feature of the mammalian amelogenetic dynamic. It might even suggest the hypothesis that the divergence of amelogenetic mechanisms into the above mentioned distinct processes could have been the most significant apomorphy of mammalian amelogenesis.

(vi) The respective processes (a,b,c) undoubtedly differ in the regulation of crystallite growth and crystallization dynamics, likely through differences in the composition of the crystallization environment. The restriction of amelotin to the surface zone and its secretion at the late stage of enamel maturation in mammals [63], suggesting a relation to process (c) mentioned above, is one example of the factors that should be taken in account. Correspondingly, a plethora of studies illustrating the role of particular components of the extracellular enamel matrix in crystallization dynamics, including supramolecular interactions producing linear aggregates of nanospheres, patterns of calcium-binding mechanisms, and inhibitory effects upon mineralization dynamics [6, 12, 19, 64], provide a robust platform for the search for pertinent regulation mechanisms.

(vii) The most detailed data on enamel formation provided by studies on incisors of muroid rodents, including high resolution TEM images of the earliest stages of crystallite formation (comp. [6]), suggest that the organization of enamel is directly promoted by the secretion activity of ameloblasts, and that prismatic and interprismatic enamel is produced simultaneously, the former by the front face, the latter by the lateral walls of the Tomes processes [65]. In contrast, our study demonstrates that the onset of mineralization both of PE and later IPM takes place in the deepest enamel zone, close to the EDJ, typically under a major cusp of the mesial part of the tooth, and subsequently spreads in the form of a maturation wave towards the surface of the crown, where the ameloblast front operates, and to the cervical region and distal part of the tooth. The essential role of the EDJ in enamel formation was repeatedly suggested also by other authors (e.g. [66]). In this regard, Simmer et al. [18] further report the demineralization of secretory products in the zone, exerting a polarizing effect upon the composition of the enamel matrix along a gradient from the ameloblast secretion front to the EDJ. In other findings, the concentration of mineral compounds along the EDJ increased together with the concentration of low molecular products of the proteolytic digestion of amelogenins and ameloblastin providing also increased amounts of calcium binding domains [25, 64, 67, 68]. It can be hypothesized that the degree of such polarization increases with the distance between the EDJ and ameloblasts, i.e. with the height of the column of prismatic enamel that, just in the porcine m3, reaches extreme values. A less obvious phenomenon indicating the essential role of EDJ in enamel formation is the conspicuous thickening of embryonic EDJ with the appearance of enamel tubules, corresponding in diameter and orientation as well as by their strongly mineralized peritubular sheets to dentine tubules (Fig 11), a character not persistent in the mature tooth. This may suggests the active role of odontoblastic processes in initiations of the prismatic enamel, possibly even providing the collagen nuclei of the prismatic sheet in the zone close to the EDJ, a phenomenon recently documented with the aid of synchrotron radiation nanotomography in two other mammalian species [69].

(viii) The contradiction between observations on muroid rodents that show prismatic and interprismatic enamel to be produced simultaneously [6] and our conclusions suggesting heterochrony in PE and IPM formation as an essential component of mammalian amelogenetic dynamics deserves special attention. It reminds us that the mode of amelogenesis in particular mammalian clades can differ considerably and that extensive adaptive divergences in dental characters can be accompanied by extensive rearrangements in amelogenetic dynamics. It should be stressed in these connections that muroid rodents, from which the vast majority of odontologic information has been obtained, exhibit an obviously extremely derived state of dental characters and extremely derived developmental dynamics, both contributing essentially to the enormous evolutionary prospect of that clade. With a complex uniserial decussation of the prisms composed of compact streams of distinct crystallites of enormous length [67], the enamel of the muroid incisor can respond, despite its minute thickness (ca 100 *μ*m in mouse), to extraordinary mechanical requirements. Obviously, during the extremely shortened developmental period of muroid rodents, such complex enamel organization could hardly be produced without radical rearrangements of the amelogenetic dynamics, shifts in the timing of particular processes and their spatial domains and/or modifications of enamel matrix proteins (note the quite derived structure of rodent amelogenin [70]). Which of the processes of enamel formation observed in muroid rodents are specific just for that clade and which of them represent true general components of mammalian amelogenetic dynamics can thus be distinguished only after detailed comparative data from other mammalian clades is available. Further information is urgently required.

## Conclusion

The prolonged calcification of the minipig distal molar (510-540 days until tooth eruption) enabled us to trace the particular stages of enamel maturation in great detail. Our results revealed that: (a) both crystallization and maturation processes start along the EDJ; (b) the pattern and mechanism of enamel formation in the early and late maturation periods differ significantly: the former is associated with the establishment of enamel prisms and the subsequent growth of their crystallites, the latter is marked by the rapid appearance of the interprismatic matrix and a shift in micromechanical properties by which, about a month prior to tooth eruption, the enamel achieves the hardeness values characterizing the adult tooth crown; (c) a rapid decrease in crystallite microstrain at the latter stage suggests that the initial organization of prismatic enamel might produce a considerable amount of structural defects that are either repaired by the subsequent growth of crystallites and/or entirely compensated by the interprismatic matrix, crystallizing at that time; and (d) early maturation producing radial prismatic enamel and late maturation associated with the appearance of IPM and resulting in adult enamel qualities should be looked upon as quite distinct processes. Their separation seems to be an essential component of mammalian amelogenetic dynamics.

## Supporting information

S1 Table Examined individuals. EdEc—expected duration of enamel calcification.
(PDF)

S2 Table List of variables. (PDF)

S3 Table Mean values and sigma statistics of particular variables under study. (PDF)

S4 Table Coeficients of Wilcoxon statistics with age as categorial variable. (PDF)

S5 Table Spearman correlation coefficients among particular variables. (PDF)

S1 Fig Selected enamel areas undergoing XRD experiments (i.e.: inner, outer, mesial, distal parts). (TIF)

S2 Fig A corellation trends of selected michromechanical properties. (TIF)

S3 Fig A cross section of post-talonid part of m3 of a16-month-old individual. note the enamel surface with no aprismatic enamel cover.
(TIF)

S4 Fig Spearman correlation plot of studied variables. (TIF)

## References

[1] Fraser GJ, Cerny R, Soukup V, Bronner-Fraser M, Streelman JT. The odontode explosion: the origin of tooth-like structures in vertebrates. Bioessays. 2010;32(9):808–817. 10.1002/bies.200900151 20730948 PMC3034446

[2] Stock DW. The genetic basis of modularity in the development and evolution of the vertebrate dentition. Philos T Roy Soc B. 2001;356(1414):1633–1653. 10.1098/rstb.2001.0917 11604128 PMC1088541

[3] Thesleff I. Epithelial-mesenchymal signalling regulating tooth morphogenesis. J Cell Sci. 2003;116(9):1647–1648. 10.1242/jcs.00410 12665545

[4] Tucker A, Sharpe P. The cutting-edge of mammalian development; how the embryo makes teeth. Nat Rev Genet. 2004;5(7):499–508. 10.1038/nrg1380 15211352

[5] Huysseune A, Sire JY, Witten PE. Evolutionary and developmental origins of the vertebrate dentition. J Anat. 2009;214(4):465–476. 10.1111/j.1469-7580.2009.01053.x 19422425 PMC2736119

[6] Nanci A. Ten Cate’s Oral Histology: Development, Structure, and Function. 8th ed Nanci A, editor. St. Louis, Missouri, USA: Mosby; 2008.

[7] Elliott JC, Holcomb DW, Young RA. Infrared determination of the degree of substitution of hydroxyl by carbonate ions in human dental enamel. Calcif Tissue Int. 1985;37(4):372–375. 10.1007/BF02553704 3930033

[8] Simmer JP, Fincham AG. Molecular mechanisms of dental enamel formation. Crit Rev Oral Biol M. 1995;6(2):84–108. 10.1177/10454411950060020701 7548623

[9] Glimcher MJ, Friberg UA, Levine PT. The isolation and amino acid composition of the enamel proteins of erupted bovine teeth. Biochem J. 1964;93(1):202–210. 10.1042/bj0930202 4953792 PMC1206201

[10] Robinson C, Lowe NR, Weatherell JA. Amino acid composition, distribution and origin of tuft protein in human and bovine dental enamel. Arch Oral Biol. 1975;20(1):29–42. 10.1016/0003-9969(75)90149-1 1054568

[11] Duverger O, Ohara T, Shaffer JR, Donahue D, Zerfas P, Dullnig A, et al Hair keratin mutations in tooth enamel increase dental decay risk. J Clin Invest. 2014;124(12):5219–5224. 10.1172/JCI78272 25347471 PMC4348957

[12] Du C, Falini G, Fermani S, Abbott C, Moradian-Oldak J. Supramolecular assembly of amelogenin nanospheres into birefringent microribbons. Science. 2005;307(5714):1450–1454. 10.1126/science.1105675 15746422

[13] Boyde A. Enamel In: Oksche A, Vollrath L, editors. Teeth. Sprimger-Verlag: Berlin; 1989 p. 309–473.

[14] Koenigswald Wv, Sander PM. Glossary of terms used for enamel microstructures In: Koenigswald Wv, Sander PM, editors. Tooth enamel microstructure. Rotterdam: A. A. Balkema; 1997 p. 267–280.

[15] Koenigswald Wv. Brief survey of enamel diversity at the schmelzmuster level in Cenozoic placental mammals In: Koenigswald Wv, Sander PM, editors. Tooth enamel microstructure. Rotterdam: A. A. Balkema; 1997 p. 137–161.

[16] Sander PM. Non-mammalian synapsid enamel and the origin of mammalian enamel prisms: the bottom-up perspective In: Koenigswald Wv, Sander PM, editors. Tooth enamel microstructure. Rotterdam: A. A. Balkema; 1997 p. 41–62.

[17] Ganss B, Abbarin N. Maturation and beyond: proteins in the developmental continuum from enamel epithelium to junctional epithelium. Front Physiol. 2014;5(1):371 10.3389/fphys.2014.00371 25309457 PMC4174742

[18] Simmer JP, Papagerakis P, Smith CE, Fisher DC, Rountrey AN, Zheng L, et al Regulation of dental enamel shape and hardness. J Dent Res. 2010;89(10):1024–1038. 10.1177/0022034510375829 20675598 PMC3086535

[19] Moradian-Oldak J. Protein-mediated enamel mineralization. Front Biosci. 2012;17(6):1996–2023. 10.2741/4034 22652761 PMC3442115

[20] Robinson C, Kirkham J, Briggs HD, Atkinson PJ. Enamel proteins: from secretion to maturation. J Dent Res. 1982;Spec.No.:1490–1495. 6958707

[21] Robinson C, Kirkham J, Brookes SJ, Bonass WA, Shore RC. The chemistry of enamel development. Int J Dev Biol. 1995;39(1):145–152. 7626401

[22] Fincham AG, Moradian-Oldak J, Simmer JP. The structural biology of the developing dental enamel matrix. J Struct Biol. 1999;126(3):270–299. 10.1006/jsbi.1999.4130 10441532

[23] Dunker AK, Lawson JD, Brown CJ, Williams RM, Romero P, Oh JS, et al Intrinsically disordered protein. J Mol Graph Model. 2001;19(1):26–59. 10.1016/S1093-3263(00)00138-8 11381529

[24] Kawasaki K, Weiss KM. SCPP gene evolution and the dental mineralization continuum. J Dent Res. 2008;87(6):520–531. 10.1177/154405910808700608 18502959

[25] Wald T, Osičková A, Šulc M, Benada O, Semerádová A, Rezábková L, et al Intrinsically disordered enamel matrix protein ameloblastin forms ribbon-like supramolecular structures via an N-terminal segment encoded by exon 5. J Biol Chem. 2013;288(31):22333–22345. 10.1074/jbc.M113.456012 23782691 PMC3829324

[26] Kirkham J, Brookes SJ, Shore RC, Bonass WA, Smith DA, Wallwork ML, et al Atomic force microscopy studies of crystal surface topology during enamel development. Connect Tissue Res. 1998;38(1-4):91–100. 10.3109/03008209809017025 11063018

[27] Robinson C, Shore RC, Wood SR, Brookes SJ, Smith DAM, Wright JT, et al Subunit structures in hydroxyapatite crystal development in enamel: implications for amelogenesis imperfecta. Connect Tissue Res. 2003;44(1):65–71. 12952176

[28] Robinson C, Yamamoto K, Connell SD, Kirkham J, Nakagaki H, Smith AD. The effects of fluoride on the nanostructure and surface pK of enamel crystals: an atomic force microscopy study of human and rat enamel. Eur J Oral Sci. 2006;114(s1):99–104. 10.1111/j.1600-0722.2006.00275.x 16674669

[29] Beniash E, Metzler RA, Lam RSK, Gilbert P. Transient amorphous calcium phosphate in forming enamel. J Struct Biol. 2009;166(2):133–143. 10.1016/j.jsb.2009.02.001 19217943 PMC2731811

[30] Cuisinier FJG, Steuer P, Senger B, Voegel JC, Frank RM. Human amelogenesis I: High resolution electron microscopy study of ribbon-like crystals. Calcif Tissue Int. 1992;51(4):259–268. 10.1007/BF00334485 1422970

[31] Daculsi G, Kerebel B. High-Resolution Electron Microscope Study of Human Enamel Crystallites: Size, Shape, and Growth. J Ultrastruct Res. 1978;65(2):163–172. 10.1016/S0022-5320(78)90053-9 731784

[32] Iijima M, Moriwaki Y, Takagi T, Moradian-Oldak J. Effects of bovine amelogenins on the crystal morphology of octacalcium phosphate in a model system of tooth enamel formation. J Cryst Growth. 2001;222(3):615–626. 10.1016/S0022-0248(00)00984-2

[33] Margolis HC, Kwak SY, Yamazaki H. Role of mineralization inhibitors in the regulation of hard tissue biomineralization: relevance to initial enamel formation and maturation. Front Physiol. 2014;5(339):1–9. 10.3389/fphys.2014.00339 25309443 PMC4159985

[34] Lu Y, Papagerakis P, Yamakoshi Y, Hu JCC, Bartlett JD, Simmer JP. Functions of KLK4 and MMP-20 in dental enamel formation. Biol Chem. 2008;389(6):695–700. 10.1515/BC.2008.080 18627287 PMC2688471

[35] Robinson C. Enamel maturation: a brief background with implications for some enamel dysplasias. Front Physiol. 2014;5 10.3389/fphys.2014.00388 25339913 PMC4189374

[36] Clemens WA. Characterization of enamel microstructure and application of the origins of prismatic structures in systematic analyses In: Koenigswald Wv, Sander PM, editors. Tooth enamel microstructure. Rotterdam: A. A. Balkema; 1997 p. 85–112.

[37] Osborn HF. Evolution of mammalian molar teeth. vol. 1 Osborn HF, editor. New York: Macmillan; 1907.

[38] Jernvall J. Mammalian Molar Cusp Patterns: Development Mechanisms of Diversity. Acta zoologica Fennica. Finnish Zoological and Botanical Pub. Board; 1995.

[39] Evans AR, Sanson GD. The tooth of perfection: functional and spatial constraints on mammalian tooth shape. Biol J Linnean Soc. 2003;78(2):173–191. 10.1046/j.1095-8312.2003.00146.x

[40] Davis BM. Evolution of the tribosphenic molar pattern in early mammals, with comments on the dual-origin hypothesis. J Mammal Evol. 2011;18(4):227–244. 10.1007/s10914-011-9168-8

[41] Lester KS, Hand SJ. Chiropteran enamel structure. Scanning Microsc. 1987;1(1):421–436. 3589613

[42] Lester KS, Boyde A. Relating developing surface to adult ultrastructure in chiropteran enamel by SEM. Adv Dent Res. 1987;1(2):181–190. 10.1177/08959374870010020601 3504168

[43] Lucas P, Constantino P, Wood B, Lawn B. Dental enamel as a dietary indicator in mammals. Bioessays. 2008;30(4):374–385. 10.1002/bies.20729 18348196

[44] Špoutil F, Vlček V, Horáček I. Enamel microarchitecture of a tribosphenic molar. J Morphol. 2010;271(10):1204–1218. 10.1002/jmor.10867 20623522

[45] Horáček I, Špoutil F. Why tribosphenic? On variation and constraint in developmental dynamics of chiropteran molars In: Gunnell GF, Simmons NB, editors. Evolutionary History of Bats: Fossils, Molecules and Morphology. 2 Cambridge University Press; 2012 p. 410–455.

[46] Štembírek J, Buchtová M, Král T, Matalová E, Lozanoff S, Míšek I. Early morphogenesis of heterodont dentition in minipigs. Eur J Oral Sci. 2010;118(6):547–558. 10.1111/j.1600-0722.2010.00772.x 21083615

[47] Tonge CH, McCance RA. Normal development of the jaws and teeth in pigs, and the delay and malocclusion produced by calorie deficiencies. J Anat. 1973;115(1):1–22. 4199500 PMC1271522

[48] Wang F, Xiao J, Cong W, Li A, Song T, Wei F, et al Morphology and chronology of diphyodont dentition in miniature pigs, Sus Scrofa. Oral Dis. 2014;20(4):367–379. 10.1111/odi.12126 23679230

[49] Klug HP, Alexander LE. Crystallite size and lattice strains from line broadening In: Klug HP, Alexander LE, editors. X-ray Diffraction procedures for polycrysstalline and amorphous materials. 2nd ed New York, USA: John Wiley and Sons, Inc.; 1974 p. 618–708.

[50] Vodička P, Smetana K, Dvořánková B, Emerick T, Xu YiZ, Ourednik J, et al The miniature pig as an animal model in biomedical research. Ann N Y Acad Sci. 2005;1049(1):161–171. 10.1196/annals.1334.015 15965115

[51] Baxa M, Hruska-Plochan M, Juhas S, Vodicka P, Pavlok A, Juhasova J, et al A transgenic minipig model of Huntington’s disease. J Huntington’s Dis. 2013;2(1):47–68. 10.3233/JHD-130001 25063429

[52] Planska D, Burocziova M, Strnadel J, Horak V. Immunohistochemical Analysis of Collagen IV and Laminin Expression in Spontaneous Melanoma Regression in the Melanoma-Bearing Libechov Minipig. Acta histochem cytochem. 2015;48(1):15 10.1267/ahc.14020 25861134 PMC4387259

[53] Kallistová A, Skála R, Horáček I, Miyajima N, Malíková R. Influence of sample preparation on the microstructure of tooth enamel apatite. J Appl Crystallogr. 2015;48(3):763–768. 10.1107/S1600576715005208

[54] Rodríguez-Carvajal J. Recent developments of the program FULLPROF. Commission on powder diffraction, Newsletter. 2001;26:12–19.

[55] Šlouf M, Vacková T, Nevoralová M, Pokorný D. Micromechanical properties of one-step and sequentially crosslinked UHMWPEs for total joint replacements. Polym Test. 2015;41(1):191–197.

[56] Oliver WC, Pharr GM. Measurement of hardness and elastic modulus by instrumented indentation: Advances in understanding and refinements to methodology. J Mater Res. 2004;19(01):3–20. 10.1557/jmr.2004.19.1.3

[57] Instruments C. 8. Software Formulas. In: Indentation Software Manual R0.1.8. CSM Instruments; 2013. p. 135–148.

[58] Daculsi G, Menanteau J, Kerebel LM, Mitre D. Lenght and Shape of Enamel Crystals. Calcif Tissue Int. 1984;36(1):550–555. 10.1007/BF02405364 6441627

[59] Robinson C, Brookes SJ, Shore RC, Kirkham J. The developing enamel matrix: nature and function. Eur J Oral Sci. 1998;106(1):282–291. 10.1111/j.1600-0722.1998.tb02188.x 9541238

[60] Sasaki S, Takagi T, Suzuki M. Cyclical changes in pH in bovine developing enamel as sequential bands. Arch Oral Biol. 1991;36(3):227–231. 10.1016/0003-9969(91)90090-H 1877895

[61] Nylen MU, Eanes ED, Omnell K. Crystal growth in rat enamel. J Cell Biol. 1963;18(1):109–123. 10.1083/jcb.18.1.109 13939321 PMC2106281

[62] Cuisinier FJG, Steuer P, Frank RM, Voegel JC. High resolution electron microscopy of young apatite crystals in human fetal enamel. J Biol Buccale. 1990;18(2):149–154. 2170347

[63] Gasse B, Chiari Y, Silvent J, Davit-Béal T, Sire JY. Amelotin: an enamel matrix protein that experienced distinct evolutionary histories in amphibians, sauropsids and mammals. BMC Evol Biol. 2015;15(1):1–16. 10.1186/s12862-015-0329-x 25884299 PMC4373244

[64] Fukumoto S, Kiba T, Hall B, Iehara N, Nakamura T, Longenecker G, et al Ameloblastin is a cell adhesion molecule required for maintaining the differentiation state of ameloblasts. J Cell Biol. 2004;167(5):973–983. 10.1083/jcb.200409077 15583034 PMC2172447

[65] Warshawsky H, Josephsen K, Thylstrup A, Fejerskov O. The Development of Enamel Structure in Rat Incisors as Compared to the Teeth of Monkey and Man. Anat Rec. 1981;200(1):371–399. 10.1002/ar.1092000402 6795971

[66] Cooper WEG. A microchemical, microradiographic and histological investigation of amelogenesis in the pig. Arch Oral Biol. 1968;13(1):46–48. 10.1016/0003-9969(68)90035-6 4867116

[67] Vymětal J, Slabỳ I, Spahr A, Vondrášek J, Lyngstadaas SP. Bioinformatic analysis and molecular modelling of human ameloblastin suggest a two-domain intrinsically unstructured calcium-binding protein. Eur J Oral Sci. 2008;116(2):124–134. 10.1111/j.1600-0722.2008.00526.x 18353005

[68] Yoshizaki K, de Vega S, Y Y. Gene evolution and functions of extracellular matrix proteins in teeth. Orthod Waves. 2013;72(1):1–10. 10.1016/j.odw.2013.01.040 23539364 PMC3607546

[69] Kallonen A, Corfe I, Hämäläinen K, Jernvall J. Three-dimensional relationships of enamel prisms, and enamel-and dentine-tubules, studied with synchrotron radiation holotomography. B Int Ass Paleodont. 2014;8(1):103.

[70] Delgado S, Girondot M, Sire JY. Molecular evolution of amelogenin in mammals. J Mol Evol. 2005;60(1):12–30. 10.1007/s00239-003-0070-8 15696365

