# Supplementary material for: Mammalian enamel maturation: Crystallographic changes prior to tooth eruption
Source: PLoS One. 2017 Feb 14;12(2):e0171424. doi: 10.1371/journal.pone.0171424 (PMC5308864; doi:10.1371/journal.pone.0171424)
Supplement: S1 Table — EdEc—expected duration of enamel calcification. (PDF) [file pone.0171424.s001.pdf]

Table S1: Examined individuals. EdEc = expected duration of enamel calcification.

**no.**	**age (month)**	**age (days)**	**EdEc (days)**
L14	16	490	250
K373	17	516	276
K291	18	553	313
K301	19	573	333
K260	20	616	376
K115	23	689	449
K283	24	731	491
K261	24	735	495
K257	24	738	498
K258	24	738	498
K41	25	747	507
K31	25	747	507
K105	26	781	541
K106	26	781	541
K113	28	857	617
G269	30	902	662
