# Supplementary material for: Mammalian enamel maturation: Crystallographic changes prior to tooth eruption
Source: PLoS One. 2017 Feb 14;12(2):e0171424. doi: 10.1371/journal.pone.0171424 (PMC5308864; doi:10.1371/journal.pone.0171424)
Supplement: S2 Table — (PDF) [file pone.0171424.s002.pdf]

Table S2: List of variables.

**variable**		**its description**
age		individual age (month)
aggregate thickness*		thickness of the core crystallite aggregates composing the radial prisms (nm)
prism width*		width of radial prisms (μm)
size	IN	mean crystallite thickness/width of inner enamel (nm)
	OUT	mean crystallite thickness/width of outer enamel (nm)
	ME	mean crystallite thickness/width of mesial tooth part (nm)
	DI	mean crystallite thickness/width of distal tooth part (nm)
strain	IN	micro-strain of inner enamel (‰)
	OUT	micro-strain of outer enamel (‰)
	ME	micro-strain of mesial tooth part (‰)
	DI	micro-strain of distal tooth part (‰)
H_{IT}	IN	indentation hardness of inner enamel (GPa)
	OUT	indentation hardness of outer enamel (GPa)
E_{IT}	IN	indentation modulus of inner enamel (GPa)
	OUT	indentation modulus of outer enamel (GPa)
C_{IT}	IN	indentation creep of inner enamel (%)
	OUT	indentation creep of outer enamel (%)
η_{IT}	IN	elastic part of indentation work of inner enamel (%)
	OUT	elastic part of indentation work of outer enamel (%)

*supplementary variable, not recorded in all individuals
