# Supplementary material for: Mammalian enamel maturation: Crystallographic changes prior to tooth eruption
Source: PLoS One. 2017 Feb 14;12(2):e0171424. doi: 10.1371/journal.pone.0171424 (PMC5308864; doi:10.1371/journal.pone.0171424)
Supplement: S3 Table — (PDF) [file pone.0171424.s003.pdf]

Table S3: Mean values and sigma statistics of particular variables under study.

**age**	**n**	**size**								**strain**							
		IN	sigma	OUT	sigma	ME	sigma	DI	sigma	IN	sigma	OUT	sigma	ME	sigma	DI	sigma
16	1	23.5	0.2	19.0	0.2	21.8	0.2	16.5	0.3	14.3	0.5	15.7	0.5	13.5	0.4	25.5	0.8
17	2	25.9	0.2	22.4	0.2	27.3	0.2	16.4	0.2	13.9	0.5	15.3	0.5	11.2	0.4	24.9	0.5
18	2	29.3	0.4	27.1	0.3	32.3	0.3	19.0	0.2	15.5	0.4	17.6	0.6	12.2	0.6	17.2	0.6
19	1	33.0	0.5	32.0	0.3	35.0	0.3	19.3	0.2	15.5	0.6	16.4	0.5	12.4	0.5	16.7	0.6
20	2	36.1	0.4	32.8	0.2	34.8	0.3	24.9	0.2	12.2	0.4	13.9	0.8	11.9	0.5	10.2	0.5
23	1	-	-	-	-	36.9	0.3	33.0	0.3	-	-	-	-	12.4	0.3	13.0	0.4
24	4	-	-	-	-	35.2	0.3	33.5	0.3	-	-	-	-	12.3	0.5	11.4	0.4
25	2	-	-	-	-	37.9	0.3	31.2	0.3	-	-	-	-	12.4	0.4	13.5	0.4
26	1	38.2	0.2	37.0	0.3	36.2	0.3	36.2	0.4	12.1	0.3	11.4	0.4	12.1	0.4	12.6	0.4
28	1	-	-	-	-	36.9	0.3	35.8	0.3	-	-	-	-	12.3	0.4	12.5	0.3
30	1	-	-	-	-	38.9	0.6	36.1	0.3	-	-	-	-	15.7	1.0	12.8	0.4

**age**	**n**	H_{IT}				E_{IT}				C_{IT}				η_{IT}			
		IN	sigma	OUT	sigma	IN	sigma	OUT	sigma	IN	sigma	OUT	sigma	IN	sigma	OUT	sigma
16	1	0.02	0.01	0.2	0.1	1.6	0.5	4.4	1.7	22.8	4.9	22.0	7.4	9.4	1.4	18.4	5
17	2	0.03	0.01	0.1	0.01	1.9	0.3	0.8	0.1	20.6	2.8	22.6	2.7	8.9	1.6	7.8	0.6
18	2	0.3	0.05	0.09	0.01	11.3	2.0	3.4	0.4	5.4	1.5	-	-	17.2	1.5	18.0	0.8
19	1	0.4	0.03	0.2	0.03	16.3	1.1	6.1	0.7	8.2	0.3	8.4	0.3	18.7	0.5	17.8	0.4
20	2	2.3	0.3	0.6	0.2	58.6	4.9	20.1	3.9	6.4	0.4	7.5	0.7	23.6	1.2	19.0	1.3
23	1	3.9	0.2	3.9	0.4	76.7	3.2	77.4	3.9	5.4	0.4	5.2	0.7	29.2	0.7	30.0	1.2
24	4	3.3	0.2	4.5	0.2	70.5	3.4	81.6	3.9	5.8	0.5	5.3	0.5	27.1	0.7	30.9	0.7
25	2	4.0	0.2	4.6	0.2	78.5	4.7	84.4	3.8	5.6	0.4	5.2	0.6	29.3	0.3	31.6	1.0
26	1	3.8	0.2	4.7	0.2	77.5	1.9	86.8	4.8	5.5	0.7	5.3	1.3	28.2	0.8	30.6	1.2
28	1	3.7	0.2	4.6	0.1	76.32	2.7	78.8	2.4	5.5	0.4	5.0	0.3	28.9	0.7	32.0	0.6
30	1	3.8	0.2	4.4	1.6	73.2	4.4	75.6	2.0	5.5	0.4	5.3	0.3	29.0	0.4	32.3	0.9
