# Supplementary material for: Mammalian enamel maturation: Crystallographic changes prior to tooth eruption
Source: PLoS One. 2017 Feb 14;12(2):e0171424. doi: 10.1371/journal.pone.0171424 (PMC5308864; doi:10.1371/journal.pone.0171424)
Supplement: S4 Table — (PDF) [file pone.0171424.s004.pdf]

Table S4: Coeficients of Wilcoxon statistics with age as categorial variable.

			**n**	**T**	**Z**	**p-value**
age	size	IN	6	0.00	2.20	0.03
		OUT	6	0.00	2.20	0.03
		ME	11	0.00	2.93	**0.003**
		DI	11	0.00	2.93	**0.003**
	strain	IN	6	0.00	2.20	0.03
		OUT	6	0.00	2.20	0.03
		ME	11	0.00	2.93	**0.003**
		DI	11	7.00	2.31	**0.002**
	H_{IT}	IN	11	0.00	2.93	**0.003**
		OUT	11	1.00	2.85	**0.004**
	E_{IT}	IN	11	10.00	2.05	0.04
		OUT	11	14.00	1.69	0.09
	C_{IT}	IN	11	3.00	2.67	**0.007**
		OUT	10	3.00	2.49	**0.01**
	η_{IT}	IN	11	31.00	0.17	0.86
		OUT	11	17.00	1.42	0.16
