# Supplementary material for: Mammalian enamel maturation: Crystallographic changes prior to tooth eruption
Source: PLoS One. 2017 Feb 14;12(2):e0171424. doi: 10.1371/journal.pone.0171424 (PMC5308864; doi:10.1371/journal.pone.0171424)
Supplement: S5 Table — (PDF) [file pone.0171424.s005.pdf]

Table S5: Spearman correlation coefficients among particular variables.

		age	size				strain				H_{IT}		E_{IT}		C_{IT}		η_{IT}	
			IN	OUT	ME	DI	IN	OUT	ME	DI	IN	OUT	IN	OUT	IN	OUT	IN	OUT
age		1.000	1.000	1.000	**0.891**	**0.936**	-0.486	-0.542	0.264	**-0.673**	**0.800**	**0.845**	**0.818**	**0.782**	-0.533	**-0.770**	**0.809**	**0.909**
size	IN	1.000	1.000	1.000	**0.943**	**0.943**	-0.486	-0.543	-0.314	**-0.943**	1.000	0.657	1.000	**0.829**	-0.657	**-0.900**	**0.943**	0.600
	OUT	1.000	1.000	1.000	**0.943**	**0.943**	-0.486	-0.543	-0.314	**-0.943**	1.000	0.657	1.000	**0.829**	-0.657	**-0.900**	**0.943**	0.600
	ME	**0.891**	**0.943**	**0.943**	1.000	**0.791**	-0.257	-0.371	0.481	-0.464	**0.918**	**0.700**	**0.873**	**0.718**	-0.579	**-0.842**	**0.945**	**0.827**
	DI	**0.936**	**0.943**	**0.943**	**0.791**	1.000	-0.429	-0.486	0.200	**-0.718**	**0.764**	**0.882**	**0.791**	**0.836**	-0.524	**-0.685**	**0.764**	**0.836**
strain	IN	-0.486	-0.486	-0.486	-0.257	-0.429	1.000	**0.943**	0.600	0.429	-0.486	-0.600	-0.486	-0.486	0.143	0.600	-0.429	-0.657
	OUT	-0.543	-0.543	-0.543	-0.371	-0.486	**0.943**	1.000	0.543	0.486	-0.543	-0.657	-0.543	-0.600	-0.029	0.600	-0.486	-0.600
	ME	0.264	-03314	-0.314	0.482	0.200	0.600	0.543	1.000	0.218	0.291	0.291	0.200	0.173	-0.123	-0.345	0.445	0.473
	DI	**-0.673**	**-0.943**	**-0.943**	-0.464	**-0.718**	0.429	0.486	0.218	1.000	-0.500	**-0.645**	-0.536	**-0.636**	0.273	0.467	-0.482	-0.600
H_{IT}	IN	**0.800**	1.000	1.000	**0.918**	**0.764**	-0.486	-0.543	0.291	-0.500	1.000	**0.773**	**0.982**	**0.845**	-0.551	**-0.806**	**0.982**	**0.727**
	OUT	**0.845**	0.657	0.657	**0.700**	**0.882**	-0.600	-0.657	0.209	**-0.645**	**0.773**	1.000	**0.845**	**0.955**	-0.333	**-0.721**	**0.764**	**0.845**
E_{IT}	IN	**0.818**	1.000	1.000	**0.873**	**0.791**	-0.486	-0.543	0.200	-0.536	**0.982**	**0.845**	1.000	**0.900**	-0.524	**-0.794**	**0.945**	**0.727**
	OUT	**0.782**	**0.829**	**0.829**	**0.718**	**0.836**	-0.486	-0.600	0.173	**-0.636**	**0.845**	**0.954**	**0.900**	1.000	-0.309	**-0.685**	**0.818**	**0.755**
C_{IT}	IN	-0.533	-0.657	-0.657	-0.579	-0.524	0.143	-0.029	-0.123	0.273	-0.551	-0.333	-0.524	-0.310	1.000	**8.999**	-0.556	-0.460
	OUT	**-0.770**	**-0.900**	**-0.900**	**-0.842**	**-0.685**	0.600	0.600	-0.345	0.467	**-0.806**	**-0.721**	**-0.794**	**-0.685**	**0.899**	1.000	**-0.842**	**-0.818**
η_{IT}	IN	**0.809**	**0.943**	**0.943**	**0.945**	**0.764**	-0.429	-0.486	0.445	-0.481	**0.982**	**0.764**	**0.945**	**0.818**	-0.556	**-0.842**	1.000	**0.791**
	OUT	**0.909**	0.600	0.600	**0.827**	**0.836**	-0.657	-0.600	0.473	-0.600	**0.727**	**0.845**	**0.727**	**0.755**	-0.460	**-0.818**	**0.791**	1.000
