# Supplementary figures and images for: Mammalian enamel maturation: Crystallographic changes prior to tooth eruption
Source: PLoS One. 2017 Feb 14;12(2):e0171424. doi: 10.1371/journal.pone.0171424 (PMC5308864; doi:10.1371/journal.pone.0171424)

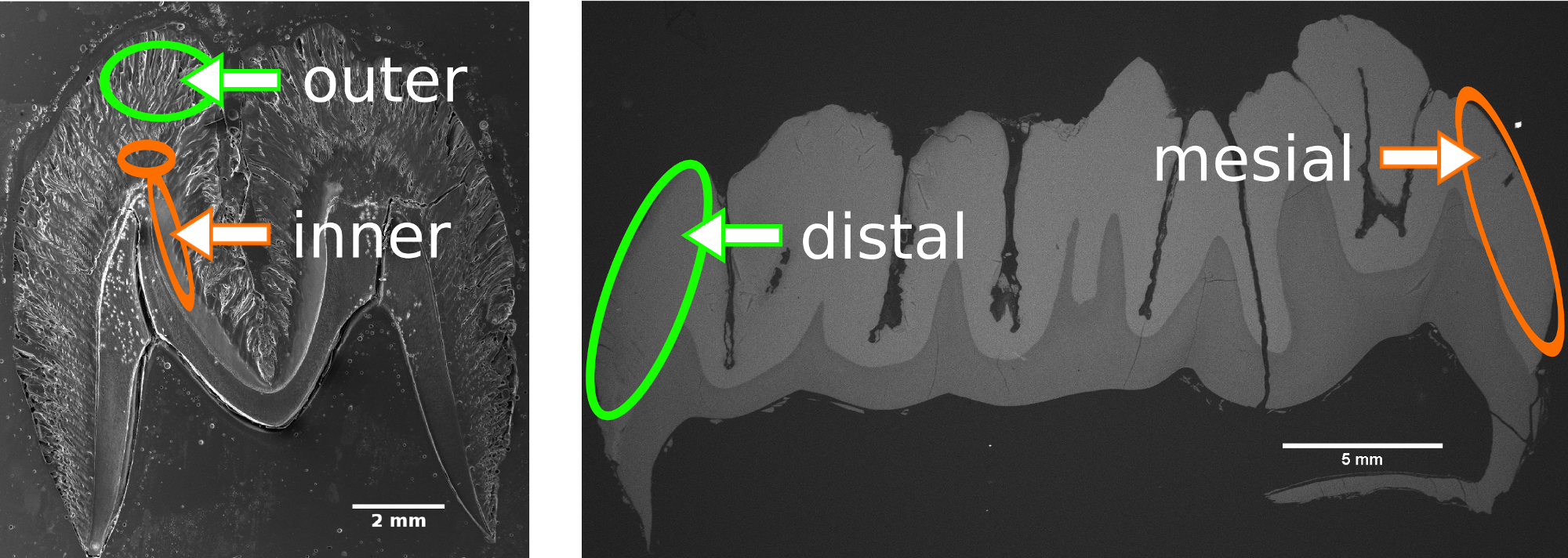

Supplement: S1 Fig — (TIF) [file pone.0171424.s006.tif]

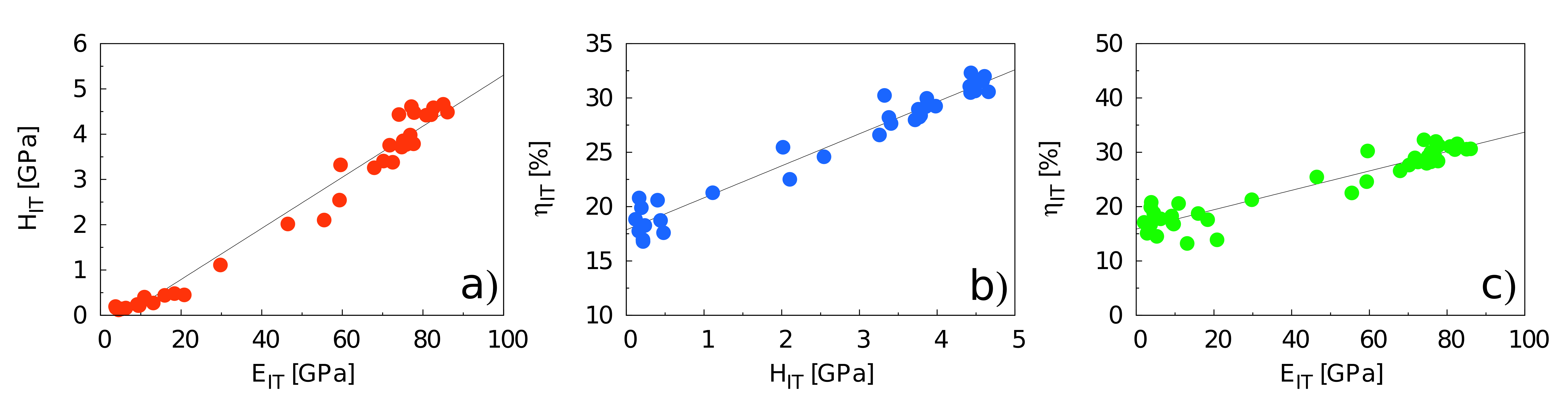

Supplement: S2 Fig — (TIF) [file pone.0171424.s007.tif]

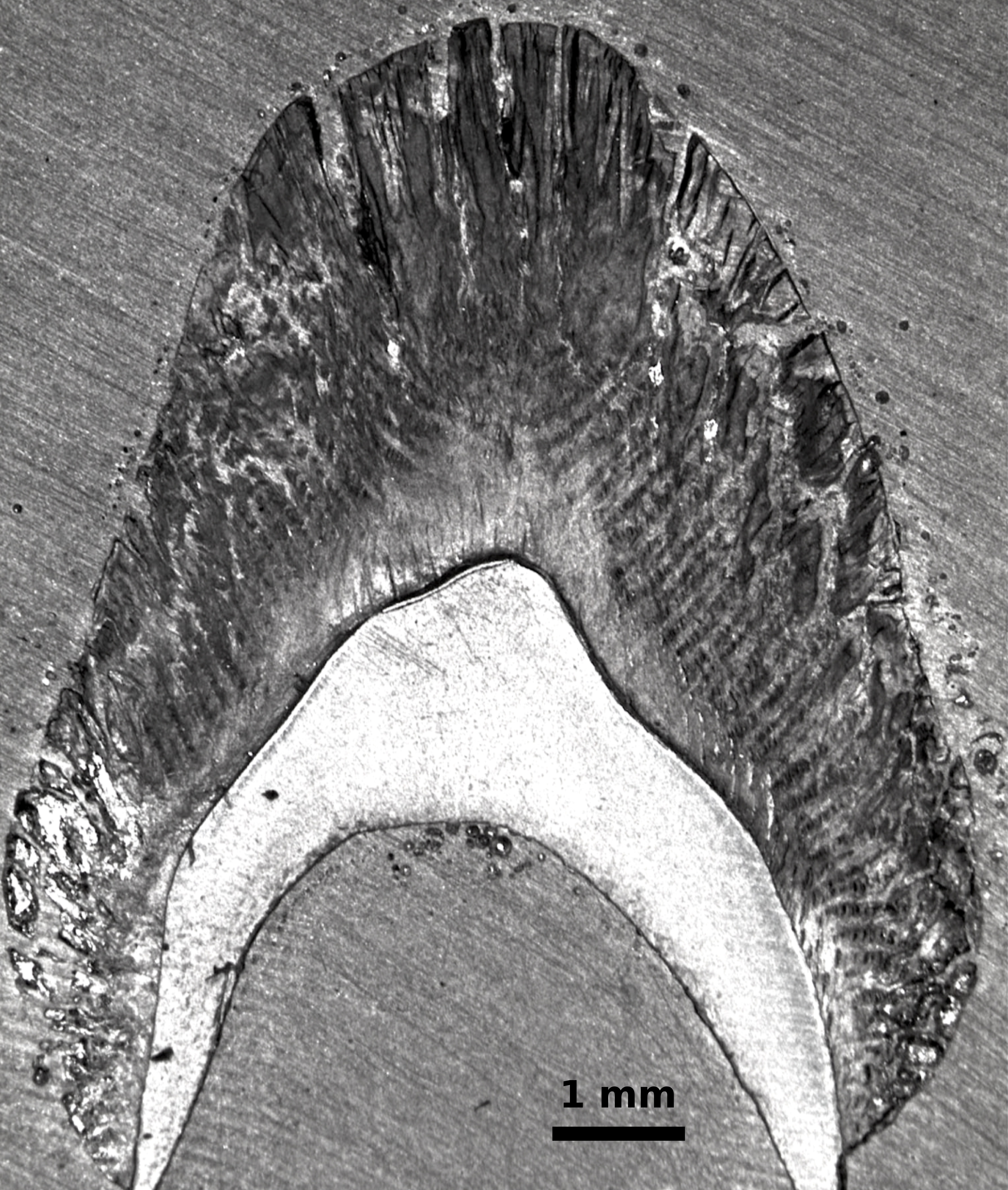

Supplement: S3 Fig — note the enamel surface with no aprismatic enamel cover. (TIF) [file pone.0171424.s008.tif]

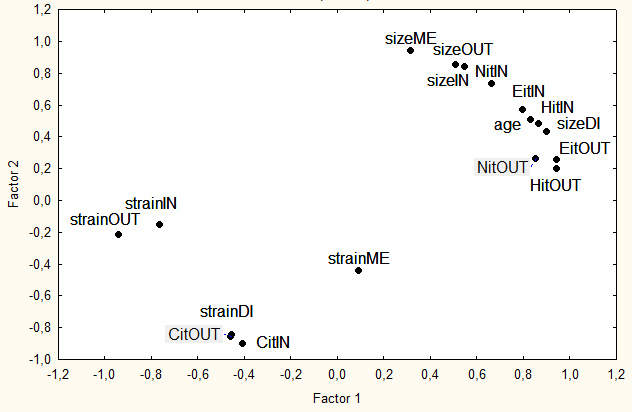

Supplement: S4 Fig — (TIF) [file pone.0171424.s009.tif]
